# Case Report: Haemolytic anaemia with ceftazidime use in a patient with cystic fibrosis

**DOI:** 10.12688/f1000research.14505.1

**Published:** 2018-04-18

**Authors:** Jun Yong, Freddy Frost, Dilip Nazareth, Martin Walshaw

**Affiliations:** 1 Liverpool Heart and Chest Hospital NHS Trust, Liverpool, L14 3PE, UK

**Keywords:** Ceftazidime, Cephalosporins, Drug Induced Immune Haemolytic Anaemia

## Abstract

Drug-induced Immune Haemolytic Anaemia (DIIHA) is a rare but serious complication of cephalosporin use. Ceftazidime is recognized to be a rare cause of DIIHA. We report and discuss a case of DIIHA in a person with cystic fibrosis who developed severe haemolytic anaemia following use of ceftazidime in the management of an acute pseudomonal pulmonary exacerbation.

## Introduction

Drug-Induced Immune Haemolytic Anaemia (DIIHA) is a serious but uncommon complication, with an estimated incidence of 1 per million of the population ^1, 2^. Since their introduction in the early 1960s, cephalosporins have been well established causes of DIIHA, most commonly with the third generation cephalosporins, cefotetan and ceftriaxone.

Ceftazidime is often a first choice cephalosporin to treat pulmonary exacerbations due to *Pseudomonas aeruginosa* in people with cystic fibrosis (CF), but despite its frequent use DIIHA is rare, with only 4 (non-fatal) cases being reported between 1971 to 2014 ^2– 4^. For the first time, we present a case of DIIHA secondary to intravenous (IV) ceftazidime use in a person with CF.

## Case report

A 23 year old female with CF (DF508/DF508) chronically infected with *Pseudomonas aeruginosa* presented with a 2 week history of general malaise, increasing shortness of breath, a productive cough and a decline in FEV _1_. She began in-patient treatment for a pulmonary exacerbation and was commenced on a 14 day course of two antipseudomonal IV antibiotics (3g of Ceftazidime and 2 Mega-Units of Colistimethate Sodium three times a day) in line with accepted CF practice. Following initial improvement, on day 4 she developed new pyrexia, hypotension, tachycardia and jaundice, and had an increasing oxygen requirement without new chest X-ray changes.

Same day laboratory investigations confirmed severe haemolytic anaemia - haemoglobin (Hb) had dropped from 127 g/L on admission to 45 g/L, with new hyper-bilirubinaemia (96 μmol/L, normal <21) and elevated Lactate Dehydrogenase (LDH) (1806 U/L, normal <250). Haptoglobin, which binds free haemoglobin in haemolysis, was undetectable at <0.1g/L (normal 0.3 – 2.0). Subsequently, she developed a reticulocytosis (145 × 10^9/L, normal range 20–110). A direct Coomb’s test was strongly positive, confirming the presence of red blood cell directed antibodies. A peripheral blood smear was negative for schistocytes and a clotting screen remained normal, thus excluding thrombotic microangiopathic processes, including disseminated intravascular coagulation and thrombotic thrombocytopenic purpura. Other investigations excluded other causes of haemolysis, including paroxysmal nocturnal haemoglobinuria, mycoplasma induced haemolysis and serum electrophoresis for lymphoproliferative disorders.

A putative diagnosis of Ceftazidime-induced DIIHA was made. Initial management included stopping the IV antibiotics and transfusing 2 units of packed red cells with parenteral steroid cover. Subsequently, oral steroids (Prednisolone 50mg/day) were commenced and continued until induction of remission. Once remission was achieved her steroids were to be tapered. As chronic haemolysis can induce folate deficiency, IV Folic Acid was also initiated. She improved significantly both clinically and biochemically, with her Hb recovering to 100 g/L within 10 days.

## Discussion

DIIHA is rare, under-recognised, and underdiagnosed, and data supporting it is poor. Often, only dramatic haemolysis leads to appropriate investigations ^2^. DIIHA is commonly associated with the presence of drug-independent and dependent antibodies.

Drug-independent antibodies are true RBC autoantibodies, targeting RBC components and not the offending drug. It is postulated that such offending drugs affect the immune system to cause RBC autoantibody formulation and subsequent haemolytic anaemia but the exact sensitization mechanism remains unknown ^2^.

Drug-dependent antibodies result from immune sensitization and generation of antibodies directed at epitopes on the drug and/or its metabolites, or a combination of drug and RBC membrane ^2, 4^. Specialized serological testing can confirm the diagnosis of DIIHA by demonstrating drug-dependent antibody induced haem-agglutination *in vitro* in the presence of the offending drug ^4^. The identification of drug-dependent antibodies could be of clinical significance, as there is evidence suggesting that patients receiving a second course of the precipitating drug or closely related drugs may develop a more severe, even fatal haemolytic anaemia due to cross-reactive antibodies ^1, 2^. Hence, patients with cephalosporin-induced haemolytic anaemia should never receive further cephalosporins.

Clinical manifestations of DIIHA lie on a spectrum ranging from mild to severe haemolysis, with life threatening complications including acute renal failure, disseminated intravascular coagulation and shock ^3^. Early identification and instigation of appropriate treatment is essential. The keystone of management is the discontinuation of the precipitant drug. Glucocorticoids (1–1.5 mg/kg/day of prednisolone or equivalent in adults) are commonly employed in inducing remission via reduction in antibody production.

Our patient’s DIIHA occurred following the 10 ^th^ dose of ceftazidime during her admission: this was her second course of this drug within two years. Rapid resolution of her haemolytic anaemia on cessation of ceftazidime with a high index of suspicion pre-treatment made serological testing for ceftazidime dependent antibodies unnecessary.

This case highlights the need for CF clinicians and microbiologists to be aware of DIIHA and its potential manifestations in order to facilitate early recognition and prompt management.

## Consent

Written informed consent for the publication of this case report was obtained from the patient.

## Data availability

All data underlying the results are available as part of the article and no additional source data are required.
