# Supplementary material for: Case Report: Haemolytic anaemia with ceftazidime use in a patient with cystic fibrosis
Source: F1000Res. 2018 Apr 18;7:475. [Version 1] doi: 10.12688/f1000research.14505.1 (PMC5920311; doi:10.12688/f1000research.14505.1)
Supplement: Peer review history

# Peer review: Referee response for version 1

**DOI:** 10.5256/f1000research.15790.r33283

**Published:** 2018-04-26

**Authors:** Charlotte Addy

**Affiliations:** 1 Centre for Experimental Medicine, Queen's University Belfast, Belfast, UK; 2 Northern Ireland Regional Adult CF Centre, Belfast City Hospital, Belfast, UK

This is a succinct and well written case report highlighting a rare but important complication of a first line anti-pseudomonal intra-venous antibiotic used commonly in Cystic Fibrosis care. It is possible this complication is under reported and without the appropriate investigations highlighted in this case may also be under-diagnosed.

 The potential additions to this case are minor. In the introduction it would be useful to clarify the details of the previous four reported cases; as it is slightly unclear whether all four cases were in individuals with CF or across other diseases without tracking down each reference. Within the discussion it would also add to the clinical relevance of the case to highlight whether drug dependent antibodies show any evidence of cross-reactivity with other similar antibiotics as is seen with hypersensitivity reactions. If there is no evidence in this regard it would be useful to state this. 

 Otherwise I am in agreement with the previous reviewer that clinical teams are alerted to this potentially significant adverse reaction.

I have read this submission. I believe that I have an appropriate level of expertise to confirm that it is of an acceptable scientific standard.

# Peer review: Referee response for version 1

**DOI:** 10.5256/f1000research.15790.r33284

**Published:** 2018-04-24

**Authors:** Jamie Duckers

**Affiliations:** 1 University Hospital Llandough, Penarth, UK

This case report details a patient with cystic fibrosis, chronically colonised with pseudomonas aeruginosa, receiving intravenous ceftazidime as part of their treatment for an acute exacerbation who develops drug induced immune haemolytic anaemia.

 Ceftazidime is often used as part of the first line intravenous antibiotic regime in patients with cystic fibrosis and those with bronchiectasis who culture pseudomonas aeruginosa. This report highlights a potentially life threatening effect of this commonly used drug for which there are no firm data on frequency but haemolytic anaemia is listed on the summary of product characteristics. I would postulate that events of drug induced immune haemolytic anaemia are under reported. It is important that clinical teams remain alert to the potential undesirable effect of this well used drug, particularly as many patients may be administering ceftazidime themselves as part of home intravenous therapy regimes to treat their acute exacerbations.

I have read this submission. I believe that I have an appropriate level of expertise to confirm that it is of an acceptable scientific standard.
